# CROSS-CULTURAL ADAPTATION OF THE LIVER DISEASE UNDERNUTRITION SCREENING TOOL TO BRAZILIAN PORTUGUESE

**DOI:** 10.1590/S0004-2803.24612026-012

**Published:** 2026-09-21

**Authors:** Marina da Silva GUEDES, Mariana Segaspini GOLOMBIESKI, Nairane Pinto BOAVENTURA, Priccila ZUCHINALI, Samanta Catherine FERREIRA, Valesca DALL ALBA

**Affiliations:** 1 Universidade Federal do Rio Grande do Sul, Faculdade de Medicina, Departamento de Nutrição, Porto Alegre, RS, Brasil.; 2 Universidade Federal do Rio Grande do Sul, Programa de Pós-Graduação em Alimentação, Nutrição e Saúde, Porto Alegre, RS, Brasil.; 3 Universidade Federal do Rio Grande do Sul, Programa de Pós-Graduação em Gastroenterologia e Hepatologia, Porto Alegre, RS, Brasil.; 4 Hospital de Clínicas de Porto Alegre, Serviço de Nutrição e Dietética, Porto Alegre, RS, Brasil.; 5 Montreal University Hospital Research Center (CRCHUM), Montreal, QC, Canada.

**Keywords:** Liver cirrhosis, nutritional screening, screening tools, triage, malnutrition, transcultural adaptation, translating, Cirrose hepática, triagem nutricional, ferramentas de triagem, triagem, desnutrição, adaptação transcultural, tradução

## Abstract

**Background:**

Chronic liver diseases represent a relevant public health problem, and undernutrition is one of their most frequent complications, being associated with higher morbidity and mortality in patients with cirrhosis. Early identification of undernutrition risk in outpatients with liver disease is fundamental; however, there is no nutritional screening tool culturally adapted for this population in Brazil. The Liver Disease Undernutrition Screening Tool (LDUST) is a simple and validated instrument for this purpose.

**Objective:**

The objective of this study was to perform the cross-cultural adaptation of the LDUST instrument into Brazilian Portuguese.

**Methods:**

The cross-cultural adaptation process followed the methodology of Beaton et al. (2000). The original tool in English was translated into Brazilian Portuguese by two independent translators, synthesized, and back-translated by two native English translators. The multidisciplinary committee evaluated semantic, idiomatic, experiential, and conceptual equivalences, consolidating the pre-final version named Triagem de Desnutrição em Pacientes com Doença Hepática (TriDH). This version was then electronically administered to healthcare professionals from different regions of Brazil. Internal reliability was analyzed using Cronbach’s alpha, adopting a minimum acceptable value of 0.8. The study was approved by the Research Ethics Committee of the Hospital de Clínicas de Porto Alegre, and all participants signed the Informed Consent Form.

**Results:**

In the pretest, the TriDH was administered to 38 healthcare professionals. It was considered easy to understand. The questionnaire presented excellent internal consistency (Cronbach’s alpha = 0.88), with all eight items exhibiting values greater than 0.8.

**Conclusion:**

The LDUST was adapted into Portuguese, originating the TriDH. This Brazilian version demonstrated high internal consistency and adequate understanding among healthcare professionals.

## HIGHLIGHTS

• The Liver Disease Undernutrition Screening Tool (LDUST) was cross-culturally adapted into Brazilian Portuguese, resulting in the TriDH instrument.

• The process followed a rigorous methodology including independent translations, back-translations, and a multidisciplinary committee review to ensure semantic and conceptual equivalence.

• The TriDH showed excellent internal consistency (Cronbach’s alpha = 0.87) and high comprehensibility among healthcare professionals nationwide.

• Illustrative images were incorporated to facilitate patient self-administration, providing a rapid and specific nutritional screening tool for outpatients with cirrhosis.

## RESUMO

**Contexto:**

As doenças hepáticas crônicas representam relevante problema de saúde pública, e a desnutrição é uma de suas complicações mais frequentes, estando associada à maior morbimortalidade em pacientes com cirrose. A identificação precoce do risco de desnutrição em pacientes ambulatoriais com doença hepática é fundamental; entretanto, não há, no Brasil, instrumento de triagem nutricional culturalmente adaptado para essa população. O Liver Disease Undernutrition Screening Tool (LDUST) é um instrumento simples e validado para esse fim.

**Objetivo:**

O objetivo deste estudo foi realizar a adaptação transcultural do instrumento LDUST para a língua portuguesa do Brasil.

**Métodos:**

O processo de adaptação transcultural seguiu a metodologia de Beaton et al. (2000). A ferramenta original em inglês foi traduzida para o português brasileiro por duas tradutoras independentes, sintetizada e retrotraduzida por dois tradutores nativos de inglês. O comitê multidisciplinar avaliou equivalências semântica, idiomática, experiencial e conceitual consolidando a versão pré-final denominada Ferramenta de Triagem de Desnutrição em Pacientes com Doença Hepática (TriDH). Esta versão foi, então, aplicada eletronicamente a profissionais de saúde de diferentes regiões do Brasil. A confiabilidade interna foi analisada pelo alfa de Cronbach, adotando-se valor mínimo aceitável de 0,8. O estudo foi aprovado pelo Comitê de Ética em Pesquisa do Hospital de Clínicas de Porto Alegre e todos os participantes assinaram o Termo de Consentimento Livre e Esclarecido.

**Resultados:**

No pré-teste, a TriDH foi aplicada a 38 profissionais de saúde. A mesma foi considerada de fácil compreensão. O questionário apresentou excelente consistência interna (alfa de Cronbach = 0,88), com todos os oito itens exibindo valores superiores a 0,8.

**Conclusão:**

A LDUST foi adaptada para a língua portuguesa, originando a TriDH. Esta versão brasileira demonstrou alta consistência interna e adequada compreensão entre profissionais de saúde.

## INTRODUCTION

Cirrhosis is a chronic liver disease (CLD) characterized by the alteration of normal liver architecture, resulting from advanced fibrosis, scarring, and the formation of regenerative nodules involving the remaining liver cells^1^. It is a progressive and severe condition, stemming from different etiologies that culminate in chronic liver inflammation. Notable among the main causes are viral hepatitis B and C, excessive alcohol consumption, and metabolic dysfunction-associated steatotic liver disease (MASLD)^2^ ^-^ ^4^.

CLD represent a major public health problem^3^, accounting for approximately two million deaths annually^4^. There has been an upward trend since 2016, currently affecting 1.3% of the world’s population^4^ ^,^ ^5^. In Brazil, although epidemiological data regarding the prevalence of cirrhosis are scarce^3^, it is estimated that 9,789 people died from the disease in 2024. The regional distribution of these occurrences reveals a higher incidence in the Southeast region, followed by the Northeast, South, Central-West, and North regions, respectively^6^. Furthermore, CLD imposes a significant burden on the healthcare system, with a high socioeconomic impact related to elevated treatment costs, prolonged periods of outpatient follow-up, and hospitalizations^7^.

Undernutrition is a common complication in patients with cirrhosis, and its presence has significant prognostic implications, as it is an independent predictor of mortality and is associated with disease decompensation and a consequent worsening of quality of life^8^. In this regard, the presence of complications, such as ascites and the development of bacterial and fungal infections, were more frequent in undernourished patients with cirrhosis compared to well-nourished ones^9^. Despite frequently affecting patients with liver disease, undernutrition remains widely underestimated and/or undetected^10^. It is diagnosed in 20% of individuals with compensated cirrhosis and 60% with decompensated cirrhosis^11^. Complications of the liver disease, such as hypoalbuminemia and ascites, may hinder the accurate detection of undernutrition^10^.

The correct identification of nutritional status in outpatients with cirrhosis is of great importance to ensure early detection and treatment of undernutrition. The use of a rapid, effective tool applicable to the evaluated population, capable of adequately identifying nutritional risk, is recommended^12^.

In this context, for the early detection of undernutrition, it is recommended to conduct screening through a tool that is simple and quick to apply, usable by professionals from various health fields or even by the patients themselves. However, it is estimated that screening is infrequently performed in outpatients with cirrhosis, likely due to the fact that, until now, there has been no low-complexity tool adapted and validated for this specific population^12^.

The Malnutrition Universal Screening Tool is an example of a validated tool indicated for outpatient use; however, it does not consider the specificities of patients with CLD. Currently, there are two tools designed for this population: Triagem Nutricional- Royal Free Hospital (TriN-RFH), which has already been cross-culturally adapted into Brazilian Portuguese but is primarily directed toward hospitalized patients^13^, and the Liver Disease Undernutrition Screening Tool (LDUST), which, until now, had not yet been adapted^12^ ^,^ ^14^ ^,^ ^15^.

The LDUST was developed based on the malnutrition consensus of the American Society for Parenteral and Enteral Nutrition (ASPEN) and the Academy of Nutrition and Dietetics (AND). The tool addresses issues related to dietary intake, weight loss, loss of body fat, muscle wasting, fluid overload, and the ability to perform daily activities. Patients classified at risk of undernutrition through this tool presented a higher risk of mortality, cirrhosis decompensation, hospitalization, and the need for emergency services^15^ ^,^ ^16^.

The LDUST identifies the presence of undernutrition in outpatients with cirrhosis^16^. For its utilization in countries other than the original location of development, a systematic process of cross-cultural adaptation is required. Therefore, the objective of this study was to perform the cross-cultural adaptation of the instrument for use in Brazil.

## METHODS

This is a cross-sectional study aiming to perform the cross-cultural adaptation to Brazilian Portuguese of the LDUST instrument, which has already been validated in English^16^. The cross-cultural adaptation process was carried out as described in the study published by Beaton et al. (2000)^17^. The project was approved by the Research Ethics Committee of the Hospital de Clínicas de Porto Alegre (HCPA) under CAAE registration number 55714719.5.0000.5327, and all participants signed the Informed Consent Form (ICF) prior to data collection.

### Step 1, forward translation

As the instrument was validated in English (United States), the first step for cross-cultural adaptation was the translation of the instrument into the target language (Portuguese). Two translators (T1 and T2) with different profiles participated in this phase; both were native Portuguese speakers proficient in English. Both performed their translations independently. T1 (P. P.) is a teacher with no knowledge of the nutrition field or the study objectives. T2 (P. Z.) is a nutritionist with prior knowledge of the subject matter and study objectives.

### Step 2, synthesis of the translations

In this phase, the translators (T1 and T2) and three researchers (N.P.B.; C.S. and V.D.A.) evaluated the translated versions to synthesize the results, producing a single version of the translation (T12).

### Step 3, back translation and synthesis

The consolidated translation (T12) was back-translated into English by two other translators (RT1: R.K.S.K. and RT2: K.J.M.B.), independently. Both were native English speakers fluent in Brazilian Portuguese and had no experience in the health field or prior knowledge of the instrument or study objectives. Subsequently, the synthesis of the back-translations (RT12) was performed by the translators (R.K.S.K. and K.J.M.B.) and researchers (N.P.B. and V.D.A.).

### Step 4, expert committee review

This stage focused on ensuring the cross-cultural equivalence of the tool. In a meeting, all translators (T1, T2, RT1, RT2), the lead author, advisor, co-advisor, and the lead author of the original tool evaluated the translated versions (T1, T2, T12, RT1, RT2, RT12). The committee’s objective was to achieve equivalence between the source and target versions in four areas: semantic, idiomatic, experiential, and conceptual equivalence. This stage resulted in the pre-final Brazilian version, named Ferramenta de Triagem de Desnutrição em Pacientes com Doença Hepática (TriDH).

### Step 5, pretesting

The TriDH was administered to a sample of healthcare professionals experienced in caring for adult patients, preferably those with liver disease. The sample size was established according to the recommendations of Beaton et al. (2000)^17^.

Contact with healthcare professionals took place electronically. The Informed Consent Form (ICF) was sent via email. All who agreed to participate in the study subsequently received the final translated version of the TriDH via an electronic form.

The eight items of the instrument were evaluated individually regarding comprehension using a Likert scale with four response categories: 1 for “I did not understand”, 2 for “I understood little”, 3 for “I understood”, and 4 for “I fully understood”. At the end of each item, there was space for suggestions and comments, which were fully transcribed and analyzed by members of the research team.

### Statistical analysis

To estimate the instrument’s reliability, internal consistency was calculated using Cronbach’s alpha, considering a minimum value of 0.8 as acceptable. Data were organized in a Microsoft Excel database, and analyses were performed using the SPSS (Statistical Package for the Social Sciences) software, version 22.0 (Chicago, IL, USA), for Windows (IBM).

## RESULTS

In the first stage, two independent versions of the instrument were created (T1 and T2). There were discrepancies between the two translations in all items of the screening tool. In the second stage, three researchers and the two translators jointly analyzed the instrument item by item, opting to retain the terms most familiar to healthcare professionals. SUPPLEMENTARY BOX 1 details the differences between the independent versions (T1 and T2), the changes made, and the synthesis with the established final version (T12).

**SUPPLEMENTARY BOX:** 1. Modifications made during the initial translation process and synthesis of the translations.

1) How have you been eating lately?	( ) Normal or fine	( ) I’ve been eating “less than normal” for a month or less	( ) I’ve been eating “less than normal” for more than one month
	( ) I’ve been trying to eat less than normal	( ) I don’t know	
T1) Quanto você tem comido ultimamente?	( ) Normal ou bem	( ) Tenho comido “menos que o normal” há um mês ou menos	( ) Tenho comido “menos que o normal” há mais de um mês
	( ) Estou tentando comer menos que o normal	( ) Não sei	
T2) Como você tem se alimentado ultimamente?	( ) Normal ou bem	( ) Eu tenho comido menos que o normal no último mês	( ) Eu tenho comido menos que o normal por mais de um mês
	( ) Eu estou tentando comer menos que o normal	( ) Eu não sei	
T12) Como você tem se alimentado ultimamente?	( ) Normal ou bem	( ) Eu tenho comido menos que o normal no último mês	( ) Eu tenho comido menos que o normal há mais de um mês
	( ) Eu estou comendo menos que o normal	( ) Eu não sei	
2) Have you lost any weight in the last year?	( ) No	( ) Yes, I have lost some weight	( ) Yes, I have lost a lot of weight
	( ) Yes, but I’ve been trying to lose weight	( ) I don’t know	
T1) Você teve perda de peso no último ano?	( ) Não	( ) Sim, tive perda de peso	( ) Sim, tive muita perda de peso
	( ) Sim, mas tenho tentado perder peso	( ) Não sei	
T2) Você perdeu peso no último ano?	( ) Não	( ) Sim, eu perdi um pouco de peso	( ) Sim, eu perdi bastante peso
	( ) Sim, mas eu venho tentando perder peso	( ) Eu não sei	
T12) Você perdeu peso no último ano?	( ) Não	( ) Sim, eu perdi peso	( ) Sim, eu perdi bastante peso
	( ) Sim, mas eu venho tentando perder peso	( ) Eu não sei	
3) Have you noticed any loss of body fat or thinning of your arms or ribs?	( ) No	( ) Yes, a little	( ) Yes, a lot
		( ) I don’t know	
T1) Você percebeu alguma perda de gordura corporal ou afinamento nos braços ou nas costelas?	( ) Não	( ) Sim, um pouco	( ) Sim, bastante
		( ) Não sei	
T2) Você notou alguma perda de gordura corporal ou redução no volume dos seus braços ou da região das costelas?	( ) Não	( ) Sim, um pouco	( ) Sim, bastante
		( ) Eu não sei	
T12) Você notou perda de gordura corporal, que seus braços estão mais finos ou suas costelas mais aparentes?	( ) Não	( ) Sim, um pouco	( ) Sim, bastante
		( ) Eu não sei	
4) Have you noticed any muscle loss in your temples, legs, clavicle, or shoulders?	( ) No	( ) Yes, a little	( ) Yes, a lot
		( ) I don’t know	
T1) Você percebeu perda de massa muscular nas têmporas, nas pernas, na clavícula ou nos ombros?	( ) Não	( ) Sim, um pouco	( ) Sim, bastante
		( ) Não sei	
T2) Você notou alguma perda de massa muscular nas suas têmporas, pernas, clavícula ou ombros?	( ) Não	( ) Sim, um pouco	( ) Sim, bastante
		( ) Eu não sei	
T12) Você notou perda de massa muscular nas têmporas (rosto), pernas, clavícula ou ombros?	( ) Não	( ) Sim, um pouco	( ) Sim, bastante
		( ) Eu não sei	
5) Do you have any fluid or swelling in your abdomen or legs?	( ) No, I have no fluid in my abdomen or legs	( ) I have some fluids in my legs or abdomen.	( ) I have a lot of fluid in my legs or abdomen.
		( ) I don’t know	
T1) Você tem alguma retenção de líquidos ou inchaço no abdômen ou nas pernas?	( ) Não, não tenho nenhum inchaço no abdômen ou nas pernas	( ) Tenho um pouco de inchaço nas pernas ou no abdômen	( ) Tenho bastante inchaço nas pernas ou no abdômen
		( ) Não sei	
T2) Você sente algum inchaço ou líquido acumulado nos seu abdômen ou nas suas pernas?	( ) Não, eu não sinto nenhum líquido acumulado no abdômen ou nas pernas	( ) Eu sinto que tenho um pouco de líquido acumulado no meu abdômen e/ou nas minhas pernas	( ) Eu sinto que tenho muito líquido acumulado no meu abdômen e/ou nas minhas pernas
		( ) Eu não sei	
T12) Você percebe acúmulo de líquido no abdômen ou inchaço nas pernas?	( ) Não, eu não percebo acúmulo de líquido no abdômen ou inchaço nas pernas	( ) Eu percebo um certo acúmulo de líquido no abdômen ou inchaço nas pernas	( ) Eu percebo bastante acúmulo de líquido no abdômen ou inchaço nas pernas
		( ) Eu não sei	
6) Are you able to participate in your usual activities? (e.g.,: walking, climbing stairs, carrying groceries)	( ) Yes, I can participate in all my usual activities	( ) No, occasionally I am too tired, weak or feel too bad to participate in my usual activities	( ) No, often I am too tired, weak or feel so bad that I cannot participate in my usual activities
		( ) I don’t know	
T1)Você consegue realizar suas atividades diárias? (Ex: caminhar, subir escadas, carregar compras)	( ) Sim, consigo realizar todas as minhas atividades	( ) Não, às vezes me sinto muito cansado(a), fraco(a), ou me sinto muito mal para realizar minhas atividades	( ) Não, frequentemente me sinto muito cansado(a), fraco(a) ou me sinto tão mal que não consigo realizar minhas atividades
		( ) Não sei	
T2) Você tem sido capaz de realizar suas atividades do dia a dia? (Ex.: caminhar, subir escadas, carregar as compras do mercado)	( ) Sim, eu tenho conseguido realizar todas minhas atividades do dia a dia	( ) Não, algumas vezes eu me sinto muito cansado(a), fraco(a) ou pouco disposto a realizar as atividades do dia a dia.	( ) Não, frequentemente eu me sinto muito cansado(a), fraco(a) ou me sinto tão indisposto que não consigo realizar as atividades do dia a dia.
		( ) Eu não sei	
T12) Você tem conseguido realizar suas atividades do dia a dia? (Ex.: caminhar, subir escadas, carregar as compras do mercado)	( ) Sim, eu tenho conseguido realizar todas minhas atividades	( ) Não, algumas vezes eu me sinto muito cansado(a), fraco(a) ou muito mal para realizar as atividades.	( ) Não, frequentemente eu me sinto muito cansado(a), fraco(a) ou tão mal que não consigo realizar as atividades
		( ) Eu não sei	
If you checked 5 or more boxes in column A: No undernutrition has been identified.		If you have checked 2 or more boxes in column B or C: Undernutrition identified. Refer for nutrition evaluation.	
T1) Se você escolheu 5 ou mais opções na coluna A: Desnutrição não identificada.a		T1) Se você escolheu 2 ou mais opções nas colunas B ou C: Desnutrição identificada. Encaminhamento para avaliação nutricional	
T2) Se você marcou 5 ou mais respostas da coluna A: Não foi identificado risco de desnutrição.		T2) Se você marcou 2 ou mais respostas das colunas B ou C: Risco de desnutrição identificado. Prossiga com avaliação nutricional completa.	
T12) Se você marcou 5 ou mais respostas da coluna A: Não foi identificado risco de desnutrição.		T12) Se você marcou 2 ou mais respostas das colunas B ou C: Risco de desnutrição identificado. Recomenda-se avaliação nutricional detalhada.	

In the third stage, version T12 was back-translated, generating two versions (RT1 and RT2). The versions showed high semantic and idiomatic equivalence, requiring only minimal vocabulary adjustments. In the fourth stage, the translators and researchers analyzed and synthesized the results, obtaining a single back-translated version (RT12). This version was sent to the original author of the tool for analysis and approval, with all modifications being approved.

In the fifth stage, the TriDH was administered to a sample of 38 healthcare professionals with experience in caring for adult patients, preferably those with liver diseases, from different regions of Brazil. Nutritionists, nurses, physicians, pharmacists, and psychologists participated. Faculty members from Nutrition and Medicine courses with experience in the field of hepatology were also included. Among the professionals, the highest participation was from nutritionists at 71.1%, followed by physicians at 21.1%; nurses, psychologists, and pharmacists represented 2.6% each.

In the pretest phase, the TriDH demonstrated excellent internal consistency (Cronbach’s alpha = 0.87). The analysis of isolated items indicated adequate corrected item-total correlations for all eight items, and the individual exclusion of any item did not result in an increase in the alpha coefficient, indicating a homogeneous contribution of the items to the internal consistency of the instrument (Table 1).

**TABLE 1:** Internal consistency analysis and item-total correlation of the TriDH tool components.

	Corrected Item-Total Correlation	Cronbach’s Alpha if Item Deleted
Item 1:	0.625	0.861
Item 2:	0.474	0.874
Item 3:	0.431	0.875
Item 4:	0.776	0.838
Item 5:	0.431	0.875
Item 6:	0.810	0.848
Item 7:	0.841	0.833
Item 8:	0.841	0.833

**Note:** the total Cronbach’s alpha coefficient for the tool was 0.87. Corrected Item-Total Correlation: indicates the degree to which each item correlates with the total scale score (higher values indicate greater correlation). Cronbach’s Alpha if Item Deleted: demonstrates what the tool’s internal consistency would be if that specific item were removed. Values lower than the total Alpha (0.87) indicate that the item’s presence contributes to increasing the tool’s reliability.

Although the tool presented high reliability, following an additional meeting among the researchers aimed at improving its applicability in the Brazilian context, a complementary step of cross-cultural adaptation was added based on the experts’ suggestions: the inclusion of illustrative images in some items to facilitate the self-administration of the instrument by the patient. These included Item 3 “Você notou perda de gordura corporal? (Ex: seus braços estão mais finos ou suas costelas mais aparentes)”, Item 4 “Você notou perda de massa muscular? (Ex: as têmporas do rosto, pernas, clavícula ou ombros mais aparentes)” and Item 5 “Você percebeu acúmulo de líquido no abdômen ou inchaço nas pernas?”.

**FIGURE 1:**
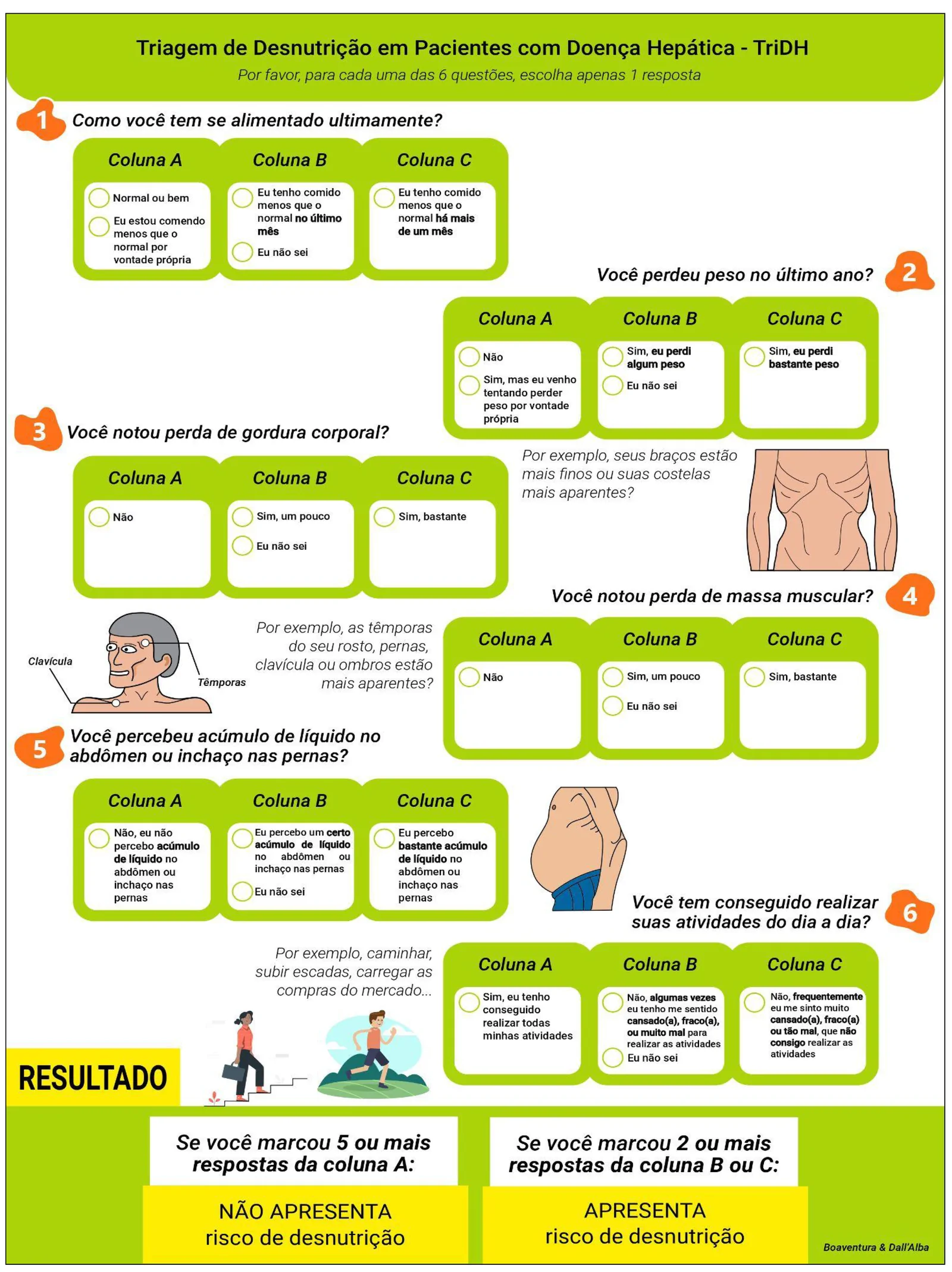
Final version of the Triagem de Desnutrição em Pacientes com Doença Hepática (TriDH).

Thus, the TriDH, the translated and cross-culturally adapted version of the LDUST to Brazilian Portuguese, was finalized, as presented in Figure 1. Terms familiar to the Brazilian clinical care context were maintained, ensuring clarity and clinical applicability. All observations reported by the professionals referred to the patients’ understanding of the terms in clinical practice and indicated that the tool was easy to understand.

## DISCUSSION

The cross-cultural adaptation of the LDUST tool into Brazilian Portuguese resulted in the TriDH, which demonstrated excellent reliability indices. Cronbach’s alpha values above 0.61 are considered indicative of substantial internal consistency, while values starting from 0.81 reflect almost perfect internal consistency^18^. Some studies consider values greater than 0.70 to be acceptable^19^. Thus, values between 0.80 and 0.90 are preferred to ensure good internal consistency^20^. In this context, the Brazilian version presented excellent psychometric performance, confirming its high reliability.

Furthermore, all items remained above 0.80, maintaining internal consistency after the cross-cultural adaptation^19^ ^,^ ^21^ ^,^ ^22^. This result demonstrates that all items comprising the tool adequately assess the risk of undernutrition in patients with cirrhosis, presenting internal correlations consistent with the evaluated construct.

The correct identification of nutritional risk in outpatients with liver disease is fundamental for establishing care priorities. Patients at higher risk are promptly referred for detailed nutritional assessment, optimizing management and outcomes^23^. In this context, the TriDH, by accounting for fluid overload, presents itself as a simple, low-cost, and rapid method developed specifically for outpatients with cirrhosis^16^. Furthermore, the authors of the original tool propose that, in addition to being administered by healthcare professionals, it can also be self-administered. This feature promotes patient autonomy and optimizes the workload of the healthcare team^15^.

The cross-cultural adaptation of the LDUST enables the use of a previously validated instrument, facilitating international comparability and resource conservation. The cultural adaptation method is a streamlined approach, as an existing instrument is made viable for use in a country other than its origin through a process that analyzes linguistic issues-moving beyond literal translation from one language to another-and evaluates the appropriate adaptation to the culture and lifestyle of the specific population. This process follows a rigorous methodology, ensuring semantic, idiomatic, conceptual, and experiential equivalencies between the versions^11^.

In the pretest phase, all eight items were easily understood by healthcare professionals. Terms familiar to healthcare professionals were retained. All observations from healthcare professionals related to the understanding of terms regarding patients treated within the reality of the public healthy system (Sistema Único de Saúde- SUS). Since the cross-cultural adaptation process aims to make the tool’s use viable in a different country, it is important to consider not only the literal translation of words from one language to another but also the set of necessary adaptations that are appropriate regarding the cultural context and lifestyle of the population served by the SUS.

Since healthcare professionals from different regions of Brazil were included, any linguistic differences were analyzed and addressed. The adaptations made sought to preserve the tool’s original design for self-administration. In question 1 (column A), for example, the phrase “por vontade própria” was added to exclude reductions in intake resulting from dietary prescriptions. To facilitate the patient’s perception of their body composition, illustrative images were inserted into questions 3 and 4, exemplifying fat loss (arms and ribs) and muscle wasting (temporal, clavicular, deltoid, and lower limb regions), respectively. Similarly, question 5 now includes visual aids to exemplify activities of daily living. Finally, the original LDUST classified patients as “no undernutrition has been identified” (for 5 or more marks in column A) or “undernutrition identified” (for 2 or more marks in column B). Considering the purpose of screening, which precedes diagnostic assessment, the terms in the TriDH were adjusted to “não apresenta risco de desnutrição” and “apresenta risco de desnutrição”.

Regarding the limitations of this study or the tool itself, previous studies indicate that the original LDUST was effective in confirming undernutrition risk, demonstrating a high positive predictive value (PPV) but with a low negative predictive value (NPV). Considering that a screening tool is expected to avoid missing subjects with potential risk, a high PPV is desirable. Conversely, a low NPV should not be interpreted as a problem, since the objective is that once risk is detected, the patient undergoes a formal assessment to confirm the finding. Another important point to consider is that the tool’s questions are patient-oriented and the responses depend on their subjective judgment, which may represent a challenge for very elderly patients or those with cognitive impairment; it is contraindicated, for example, in more severe cases of encephalopathy. To compensate for this limitation, the questions may also be directed to a family member or caregiver, and the healthcare professional can assist whenever necessary to facilitate understanding^12^ ^,^ ^14^.

The present study considers Brazil in its continental dimension, with different regions; thus, the researchers ensured the inclusion of evaluators from different Brazilian regions. This study involved healthcare professionals from Rio Grande do Sul, Paraná, São Paulo, Rio de Janeiro, Minas Gerais, and Bahia, broadening the generalizability of the findings.

Thus, the LDUST nutritional screening tool was culturally adapted to Brazilian Portuguese, originating the TriDH, which demonstrated high reliability. There is potential for its use in health services attending outpatients with liver diseases in the country. The next step consists of validating the instrument for use in clinical practice among Brazilian outpatients. 

## CONCLUSION

The cross-cultural adaptation of the LDUST instrument was conducted according to internationally accepted and well-established methodology, resulting in the TriDH. In the pretest, this Brazilian version demonstrated high reliability and proved to be easily understood by healthcare professionals. Expert evaluations confirmed the conceptual equivalence and clarity of the items, requiring only minor adaptations to facilitate patient understanding.

Thus, the TriDH presents high reliability for detecting nutritional risk in Brazilian patients with liver diseases. However, the continuation of validation stages is necessary to ensure its diagnostic accuracy and routine applicability in clinical practice.

## Data Availability

Data-available-upon-request

## References

[1] Nusrat S, Khan MS, Fazili J, Madhoun MF (2014). Cirrhosis and its complications: evidence based treatment. World J Gastroenterol.

[2] Tilg H, Petta S, Stefan N, Targher G (2025). Metabolic Dysfunction-Associated Steatotic Liver Disease in Adults: A Review. JAMA.

[3] de Carvalho JR, Villela-Nogueira CA, Perez RM, Portugal FB, Flor LS, Campos MR (2017). Burden of Chronic Viral Hepatitis and Liver Cirrhosis in Brazil - the Brazilian Global Burden of Disease Study. Ann Hepatol.

[4] Devarbhavi H, Asrani SK, Arab JP, Nartey YA, Pose E, Kamath PS (2023). Global burden of liver disease: 2023 update. J Hepatol.

[5] Zamani M, Alizadeh-Tabari S, Ajmera V, Singh S, Murad MH, Loomba R (2025). Global Prevalence of Advanced Liver Fibrosis and Cirrhosis in the General Population: A Systematic Review and Meta-analysis. Clin Gastroenterol Hepatol.

[6] Brasil. Ministério da Saúde (2025). Sistema de Informações sobre Mortalidade - SIM.

[7] Rakoski M, Goyal P, Spencer-Safier M, Weissman J, Mohr G, Volk M (2018). Pain management in patients with cirrhosis. Clin Liver Dis.

[8] Moctezuma-Velázquez C, García-Juárez I, Soto-Solís R, Hernández-Cortés J, Torre A (2013). Nutritional assessment and treatment of patients with liver cirrhosis. Nutr Burbank Los Angel Cty Calif.

[9] Pérez-Reyes E, Rivera-Sánchez J, Servín-Caamaño AI, Pérez-Torres E, Abdo-Francis JM, Higuera-de La Tijera F (2016). Malnutrition is related to a higher frequency of serious complications in patients with cirrhosis. Rev Médica Hosp Gen México.

[10] Borhofen SM, Gerner C, Lehmann J, Fimmers R, Görtzen J, Hey B (2016). The Royal Free Hospital-Nutritional Prioritizing Tool Is an Independent Predictor of Deterioration of Liver Function and Survival in Cirrhosis. Dig Dis Sci.

[11] Singal AK, Wong RJ, Dasarathy S, Abdelmalek MF, Neuschwander-Tetri BA, Limketkai BN (2025). ACG Clinical Guideline: Malnutrition and Nutritional Recommendations in Liver Disease. Off J Am Coll Gastroenterol ACG.

[12] Tandon P, Raman M, Mourtzakis M, Merli M (2017). A practical approach to nutritional screening and assessment in cirrhosis. Hepatol Baltim Md.

[13] Glasenapp JH, Zuchinali P, Alba VD (2023). Translation and cross-cultural adaptation of the Royal Free Hospital- Nutritional Prioritizing Tool (RFH-NPT). Arq Gastroenterol.

[14] Ney M, Li S, Vandermeer B, Gramlich L, Ismond KP, Raman M (2020). Systematic review with meta-analysis: Nutritional screening and assessment tools in cirrhosis. Liver Int.

[15] Casas-Deza D, Bernal-Monterde V, Betoré-Glaria E, Julián-Gomara AB, Yagüe-Caballero C, Sanz-París A (2023). Liver Disease Undernutrition Screening Tool Questionnaire Predicts Decompensation and Mortality in Cirrhotic Outpatients with Portal Hypertension. Nutrients.

[16] Booi AN, Menendez J, Norton HJ, Anderson WE, Ellis AC (2015). Validation of a Screening Tool to Identify Undernutrition in Ambulatory Patients With Liver Cirrhosis. Nutr Clin Pract.

[17] Beaton D, Bombardier C, Guillemin F, Ferraz MB (2000). Guidelines for the Process of Cross-Cultural Adaptation of Self-Report Measures. Spine.

[18] Landis JR, Koch GG (1977). The measurement of observer agreement for categorical data. Biometrics.

[19] Souza AC, Alexandre NMC, Guirardello EB (2017). Propriedades psicométricas na avaliação de instrumentos: avaliação da confiabilidade e da validade. Epidemiologia e serviços de saúde.

[20] Almeida D, dos Santos MAR, Costa AFB (2010). Aplicação do Coeficiente Alfa de Cronbach nos resultados de um questionário para avaliação de desempenho da saúde pública.

[21] Wei T, Ying C, Oksan B (2014). Internal Consistency: Do We Really Know What It Is and How to Assess It?. J Psychol Behav Sci.

[22] Fortes CPDD, Araújo APDQC (2019). Check list para tradução e Adaptação Transcultural de questionários em saúde. Cad Saúde Coletiva.

[23] Saueressig C, Glasenapp JH, Luft VC, Alves FD, Ferreira PK, Hammes TO (2020). Phase Angle Is an Independent Predictor of 6-Month Mortality in Patients With Decompensated Cirrhosis: A Prospective Cohort Study. Nutr Clin Pract Off Publ Am Soc Parenter Enter Nutr.

